# Complete mitochondrial genome of *Lycodes tanakae* (Perciformes: Zoarcidae)

**DOI:** 10.1080/23802359.2016.1261613

**Published:** 2017-01-09

**Authors:** Yung Kun Kim, Young Sun Song, Il-Hun Kim, Chung-Bae Kang, Won Bi Kim, Seong-Yong Kim

**Affiliations:** a National Institute of Ecology, Seocheon-gun, Chungcheongnam-do, South Korea;; b National Marine Biodiversity Institute of Korea, Seocheon-gun, Chungcheongnam-do, South Korea

**Keywords:** *Lycodes tanakae*, Zoarcidae, mitochondria genome

## Abstract

The complete mitochondrial genome of *Lycodes tanakae* was sequenced for the first time from its muscle tissue using the next-generation sequencing method. Its mitochondrial genome was 16,594 base pairs in length, containing 13 protein-coding genes, 22 transfer RNA genes, two ribosomal RNA genes, and one control region. Its overall A, C, G, and T contents were 25.6%, 30.6%, 18.7%, and 25.2%, respectively. Its, A + T content (50.8%) was slightly higher than its G + C content (49.2%). A phylogenetic tree was built using 10 belonging to the order Perciformes and two species belonging to the order Scorpaeniformes.

*Lycodes tanakae* Jordan and Thompson 1914 belongs to Perciform family Zoarcidae. They are known as benthic fishes with distribution in the East Sea, Northern part of Japan, and Russia (Jordan & Thomson 1914; Mori 1952; Sokolovskaya et al. 1998; Kim et al. 2005; Nakabo 2013). *Lycodes tanakae* (Jang-chi in Korean) is a commercially valuable fish species (Choi et al. 2013).

In the present study, *L*. *tanakae* specimen was collected from a commercial fish market (Mukho-port, Donghae-si) near the East Sea of Korea. The specimen was deposited at the National Marine Biodiversity Institute of Korea (MABIK PI00039362). We dissected the right dorso-lateral muscle of the specimen and preserved it in 95% ethanol. Genomic DNA was extracted from the sample using Qiagen DNeasy Blood and Tissue kit (Qiagen Korea Ltd, Seoul, South Korea) following the manufacturer’s instructions. The complete mitochondrial DNA was sequenced on a Hiseq2000 platform using next-generation sequencing (NGS) technique (Illumina, San Diego, CA). Geneious 9.1.3 (Biomatters Ltd, Auckland, New Zealand), tRNA Scan-SE1.21 software (http://lowelab.ucsc.edu/tRNA Scan-SE/), and MitoFish (Mitochondrial Genome Database of Fish, http://mitofish.aori.u-tokyo.ac.jp/) were used to assemble and annotate the mitochondrial DNA sequences.

The complete mitochondrial genome (GenBank Accession No. KX148472) was 16,594 base pairs in length. Its overall A, C, G, and T contents were, 25.6%, 30.6%, 18.6%, and 25.2%, respectively. Its A-T content (50.8%) was slightly higher than its G-C content (49.2%). This genome contained 13 protein-coding genes (*ND1-ND6*, *ND4L*, *COI-COIII*, *ATP6*, *ATP8*, and *CYTB*), 2 rRNA genes (*12s* and *16s RNA*), 22 tRNA genes, and 1 D-loop region. Of protein-coding genes, the longest one was *ND5* (1839 bp) and the shortest one was *ATP8* (168 bp). The 12S and 16S rRNA genes were 946 bp and 1692 bp in length, respectively. They were interspaced by the tRNA gene *tRNA-Val*. Most genes of the mitogenome of *L. tanakae* were encoded on the L-strand except for the *ND6* and *tRNA* (Gln, Ala, Asn, Cys, Tyr, Ser (UCN), Glu and Pro) genes which were encoded on the H-strand. All 13 protein-coding genes had the same start codon ATG except gene *COI* which had GTG as the start codon instead. However, the stop codons of the 13 protein-coding genes varied. Among the 13 protein-coding genes, four genes (*COI*, *ATP8*, *ND4L*, and *ND6*) had a stop codon of TAA, while *ND1* and *ND5* had a stop codon of TAG. *ATP5* and *COIII* ended with TA– while and five genes (*ND2*, *COII*, *ND3*, *ND4*, and *CYTB*) ended with T––. These results might help elucidate further phylogenetic relationship among different species of Zoarcidae. The order and pattern of gene arrangement in this mitogenome were identical to those of other species in genus *Lycodes* (Miya et al. 2003, GenBank Accession No. AP004448, *L. toyamensis*, Figure 1). The mitogenome sequence of *L. tanakae* will provide useful information for understanding the evolutionary history and phylogenetic relationship for genus *Lycodes* within the family Zoarcidae.

**Figure 1.**
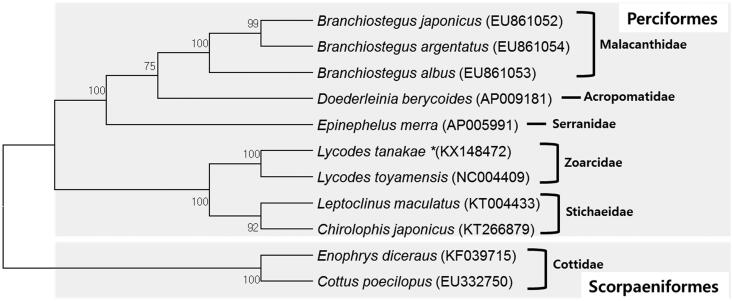
A maximum parsimony (MP) tree using coding genes of complete mitochondrial genomes of *L. tanakae* and 10 species belonging to order Perciformes as well as 2 species belonging to order Scorpaeniformes. The complete mitogenomes were downloaded from GenBank (accession number shown after the scientific name of each species). The phylogenetic tree was constructed with MEGA 6 using 1000 bootstrap replicates.
